# Relevance of *Candidatus* Nitrotoga for nitrite oxidation in technical nitrogen removal systems

**DOI:** 10.1007/s00253-021-11487-5

**Published:** 2021-09-11

**Authors:** Eva Spieck, Simone Wegen, Sabine Keuter

**Affiliations:** Department of Microbiology and Biotechnology, Universität Hamburg, Hamburg, Germany

**Keywords:** Nitrification, Nitrite oxidizing bacteria, *Cand.* Nitrotoga, Environmental distribution, Selective factors

## Abstract

**Abstract:**

Many biotechnological applications deal with nitrification, one of the main steps of the global nitrogen cycle. The biological oxidation of ammonia to nitrite and further to nitrate is critical to avoid environmental damage and its functioning has to be retained even under adverse conditions. Bacteria performing the second reaction, oxidation of nitrite to nitrate, are fastidious microorganisms that are highly sensitive against disturbances. One important finding with relevance for nitrogen removal systems was the discovery of the mainly cold-adapted *Cand*. Nitrotoga, whose activity seems to be essential for the recovery of nitrite oxidation in wastewater treatment plants at low temperatures, e.g., during cold seasons. Several new strains of this genus have been recently described and ecophysiologically characterized including genome analyses. With increasing diversity, also mesophilic *Cand.* Nitrotoga representatives have been detected in activated sludge. This review summarizes the natural distribution and driving forces defining niche separation in artificial nitrification systems. Further critical aspects for the competition with *Nitrospira* and *Nitrobacter* are discussed. Knowledge about the physiological capacities and limits of *Cand.* Nitrotoga can help to define physico-chemical parameters for example in reactor systems that need to be run at low temperatures.

**Key points:**

• *Characterization of the psychrotolerant nitrite oxidizer Cand. Nitrotoga*

• *Comparison of the physiological features of Cand. Nitrotoga with those of other NOB*

• *Identification of beneficial environmental/operational parameters for proliferation*

**Supplementary Information:**

The online version contains supplementary material available at 10.1007/s00253-021-11487-5.

## Introduction

Nitrification is the biological oxidation of ammonia to nitrate in two steps, performed by highly specialized ammonia oxidizing bacteria/archaea (AOB/AOA) and nitrite oxidizing bacteria (NOB). This process is of environmental importance to avoid accumulation of harmful ammonia and nitrite, which can result in human and aquatic animal health risk (Camargo and Alonso 2006). Furthermore, the endproduct of nitrification–nitrate-causes eutrophication of effluent-receiving waters from wastewater treatment plants (WWTPs), but can be reduced to nitrous oxide and molecular nitrogen by nitrifying and heterotrophic denitrifying bacteria. Therefore, nitrification is essential to remediate excessive N-nutrients from sewage and contributes to global nitrous oxide emissions.

**Fig. 1:**
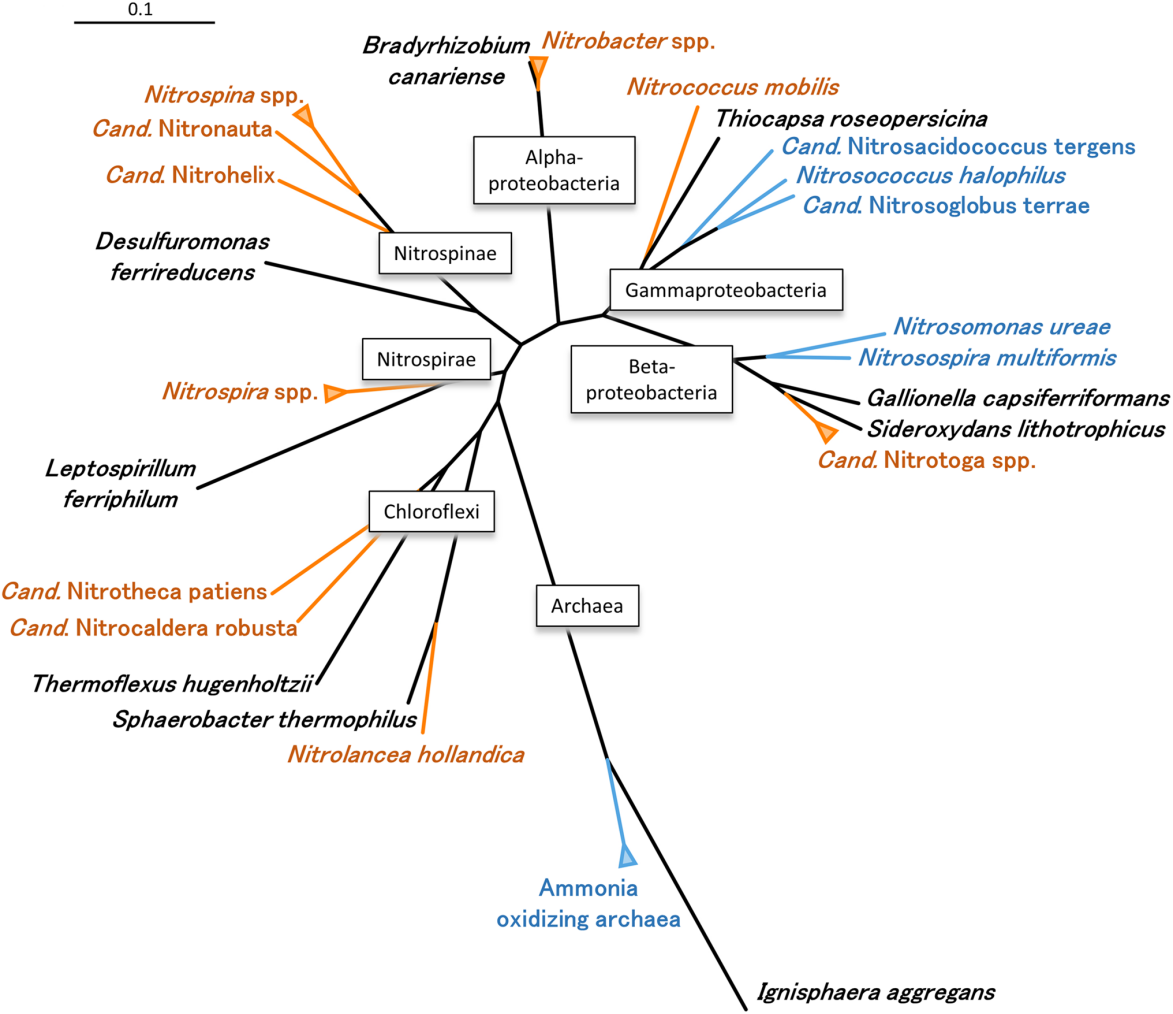
Phylogenetic tree (NJ) based on 16S rRNA genes showing the relationships of known genera of nitrite oxidizing bacteria (in red) in their respective phylum/class between each other, with closely related non-nitrifying bacteria (in black) and ammonia oxidzing bacteria/archaea (in blue). The scalebar indicates 0.1 estimated nucleotide substitutions per site

In engineered systems, ammonia oxidation, the first step of nitrification, is mainly performed by *Betaproteobacteria* of the genus *Nitrosomonas* (Koops and Pommerening-Röser 2001). In contrast, NOB, which consume the product of ammonia oxidation, are a very heterogenous group and their members are spread over the phylogenetic tree (Fig. 1). *Nitrospira* is considered as key NOB in municipal and industrial WWTPs (Daims et al. 2006; Wu et al. 2019) with a high phylogenetical and metabolic diversity (Pester et al. 2014; Koch et al. 2015). The awareness of this NOB increased once again with the discovery of comammox *Nitrospira*, combining ammonia and nitrite oxidation in a single cell (Daims et al. 2015; Van Kessel et al. 2015). Although research on nitrite oxidation has a long tradition in microbiology, an impressive taxonomic and physiological diversity was uncovered only within the last few years (Koch et al. 2014, 2015; Daims et al. 2016; Spieck et al. 2020a, b; Mueller et al. 2021).

**Fig. 2:**
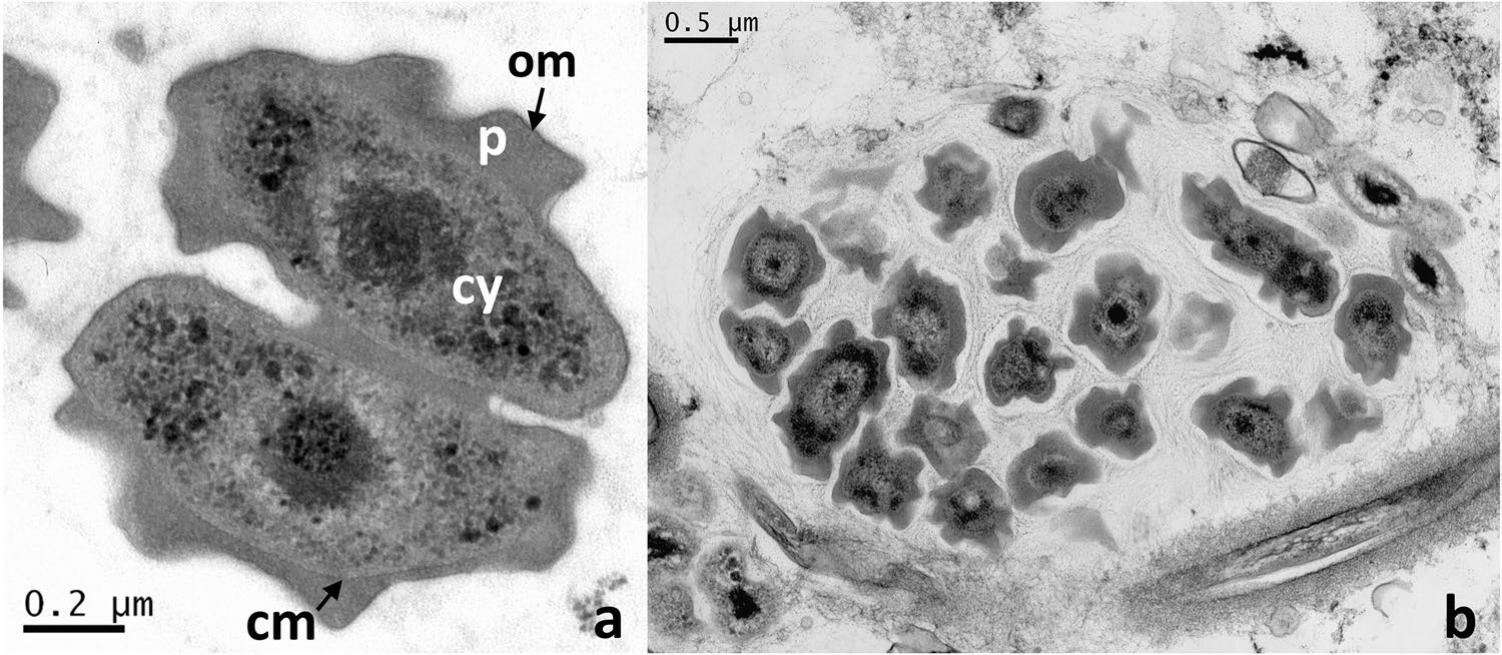
Electron micrographs of ultrathin sections of *Cand.* Nitrotoga cells revealing the characteristic ultrastructure. **a** *Cand.* N. arctica in division, with the extraordinary wide periplasmic space of particulate nature. **b** Microcolony of *Cand*. Nitrotoga with cells surrounded by thin layers of EPS. cm = cytoplasmic membrane, p = periplasm, om = outer membrane, cy = cytoplasm

Only a few years ago, the nitrite-oxidizing genus *Cand.* Nitrotoga was discovered among nitrifiers in permafrost-affected soil from Samoylov Island in the Lena Delta (Alawi et al. 2007). Diverse microbial communities are able to survive the harsh environmental conditions and *Cand.* Nitrotoga coexists with *Nitrospira* and *Nitrobacter* in this N-limited ecosystem (Wagner et al. 2001; Sanders et al. 2019). No representative of the latter two NOB was cultivated at low temperatures and solely *Cand.* Nitrotoga arctica could be enriched when incubation temperatures between 4 and 17 °C were used, which are lower than the in situ maximum temperature of Siberian soil during summer. Cells of *Cand.* Nitrotoga are irregularly shaped short rods or cocci, which are characterized by a distinct ultrastructure with an unusually wide periplasmic space, hence the name “toga” (Alawi et al. 2007; Fig. 2a). Like other NOB, they live in microcolonies, but the biofilm formation is less pronounced than in *Nitrospira* or *Nitrobacter* (Fig. 2b). The individuals are connected by a loose structure of extracellular polymeric substances (EPS) in small aggregates, which can occur in close vicinity to AOB (Lücker et al. 2015). Phylogenetically, the cold-adapted NOB belongs to the *Betaproteobacteria-*like ammonia oxidizing bacteria (*Nitrosomonas*, *Nitrosospira*) (Fig. 1).

Since the description of *N. arctica* in 2007, an increasing number of *Cand.* Nitrotoga representatives have been detected in natural as well as engineered ecosystems. Successful enrichments were initiated with samples from permafrost soil, activated sludge, freshwater and marine biofilters, rivers, coastal sediment, and the terrestrial subsurface (Alawi et al. 2007, 2009; Boddicker and Mosier 2018; Hüpeden et al. 2016; Ishii et al. 2017; Keuter et al. 2017; Kitzinger et al. 2018; Wegen et al. 2019).

Typical for most NOB, growth of *Cand.* Nitrotoga with nitrite as substrate is slow with generation times of 44–54 h (Nowka et al. 2015a; Ishii et al. 2017). To date, there are only two pure cultures available (Kitzinger et al. 2018; Ishii et al. 2020), and as described for *Nitrospira* (Nowka et al. 2015b), isolation is time-intensive and requires a combination of several methods, mainly to eliminate accompanying heterotrophic bacteria that adhere to and use organic material excreted by the NOB. Furthermore, growth of the NOB might benefit or even require the exchange of metabolites or other cofactors like ammonium (Wegen et al. 2019; Ishii et al. 2020) in coculture with heterotrophs. *Cand.* Nitrotoga could be frozen by a special cryopreservation method (Vekeman et al. 2013), but reactivation of stored cultures is less successful than for other NOB and deposition in bacterial culture collections was not possible due to the extensive need of manpower. As a consequence, no strain has been validly described and *Nitrotoga* is still a *Candidatus* taxon (Oren et al. 2020).

Notably, since *Cand.* Nitrotoga was not classified by the RDP database (Navada et al. 2020a), it can be—and possibly often was—easily overlooked in 16S rRNA-based analyses. Therefore, to target these NOB in studies of N-removal systems, appropriate methods should be used.

## Occurrence in natural habitats

The environmental distribution of *Cand.* Nitrotoga-related 16S rRNA gene sequences confirmed that many of these NOB are associated with low temperatures in extreme environments. However, closely related 16S rRNA gene sequences were not restricted to cold climates, but were found to be globally distributed from the tropics to the poles over a wide range of temperatures. A search against sequences in the NCBI Sequence Read Archive found that sequences of these NOB were detected mainly in samples from soils, wastewater, sediments, and freshwater, and in some of the samples, a temperature of about 30 °C was measured (Boddicker and Mosier 2018).

Polar regions are a typical habitat for *Cand.* Nitrotoga (Alawi et al. 2007; Achberger et al. 2016; Kohler et al. 2020) and, for example, in the subglacial Lake Whillans in the West Antarctic, it was among the most abundant bacteria and the only NOB (Christner et al. 2014). In this deep cold freshwater habitat, which is covered by an 800-m ice sheet, nitrification is one of the drivers for primary production to sustain microbial life. Accordingly, *Cand.* Nitrotoga was also found in high-elevated (peri)glacial soils and they were likely involved in nitrification in soils exposed to extreme freeze–thaw cycles (Pradhan et al. 2010; Schmidt et al. 2009). Strong temperature fluctuations were also measured in annual cycles of a seasonally ice-covered river in Canada. Here, the abundance of *Cand.* Nitrotoga increased in the late winter season in correlation with a rise in the nitrogen concentration (Cruaud et al. 2020). In a subalpine peatland in China, *Cand.* Nitrotoga was identified as one of a few keystone species of the bacterial communities (Tian et al. 2020) and it was the most abundant nitrifier in cryoconite granules on glacier surfaces in China (Segawa et al. 2020).

Further natural habitats for *Cand.* Nitrotoga are temperate freshwater, groundwater, or CO_2_-rich mineral water (Krauze et al. 2017). High abundances were found in an ice-covered Canadian lake (Fournier et al. 2021) and in the Laurentian great lakes (Paver et al. 2020). Four different *Cand.* Nitrotoga cultures were enriched from urban- or agriculturally impacted rivers in CO, USA (Boddicker and Mosier 2018), and it was also detected in the tidal reach of the Yangtze river (Fan et al. 2016). Additionally, different kinds of filter systems used for the production of drinking water provided a suitable surface to enrich high cell numbers of *Cand.* Nitrotoga reaching about 20% of the 16S rRNA gene sequences (Kaarela et al. 2015; Cai et al. 2016; Albers et al. 2018; White et al. 2012; Table S1). In drinking water distribution systems, *Cand.* Nitrotoga coexisted together with *Nitrospira* and *Nitrobacter* and its abundance increased when the water was disinfected with chloramine (Waak et al. 2019).

Additionally, *Cand.* Nitrotoga is a main NOB involved in primary production driven by geochemical processes in caves and subsurface soils. Its activity was revealed in the sulfur- and ammonium-based chemolithotrophy in the Movile cave, Romania (Chen et al. 2009), and a strain was enriched from samples of the Äspö Hard Rock Laboratory, Sweden (Keuter et al. in preparation). It became obvious that *Cand.* Nitrotoga often occurs in Fe-based microbial ecosystems, e.g., in a groundwater seep (Roden et al. 2012), the Sitarjevec Mine, Slovenia (Toplak et al. 2021), the mentioned Äspö Hard Rock Laboratory (Ionescu et al. 2015) or Fe-rich paddy soil (Naruse et al. 2019). Interestingly, the next related taxonomically described bacteria are the iron oxidizers *Gallionella ferruginea* and *Sideroxydans lithotrophicus*. Comparative genomics of *Cand.* Nitrotoga from river sediment and water column samples revealed an array of genes for iron acquisition, which may offer a competitive advantage in iron-limited environments, but siderophores were not present (Boddicker and Mosier 2018). As iron-sulfur centers are involved in the transformation of nitrite to nitrate (Meincke et al. 1992), the availability of this element is crucial to maintain the energy delivering reaction active.

## *Cand.* Nitrotoga in engineered ecosystems

### Wastewater treatment plants

**Fig. 3:**
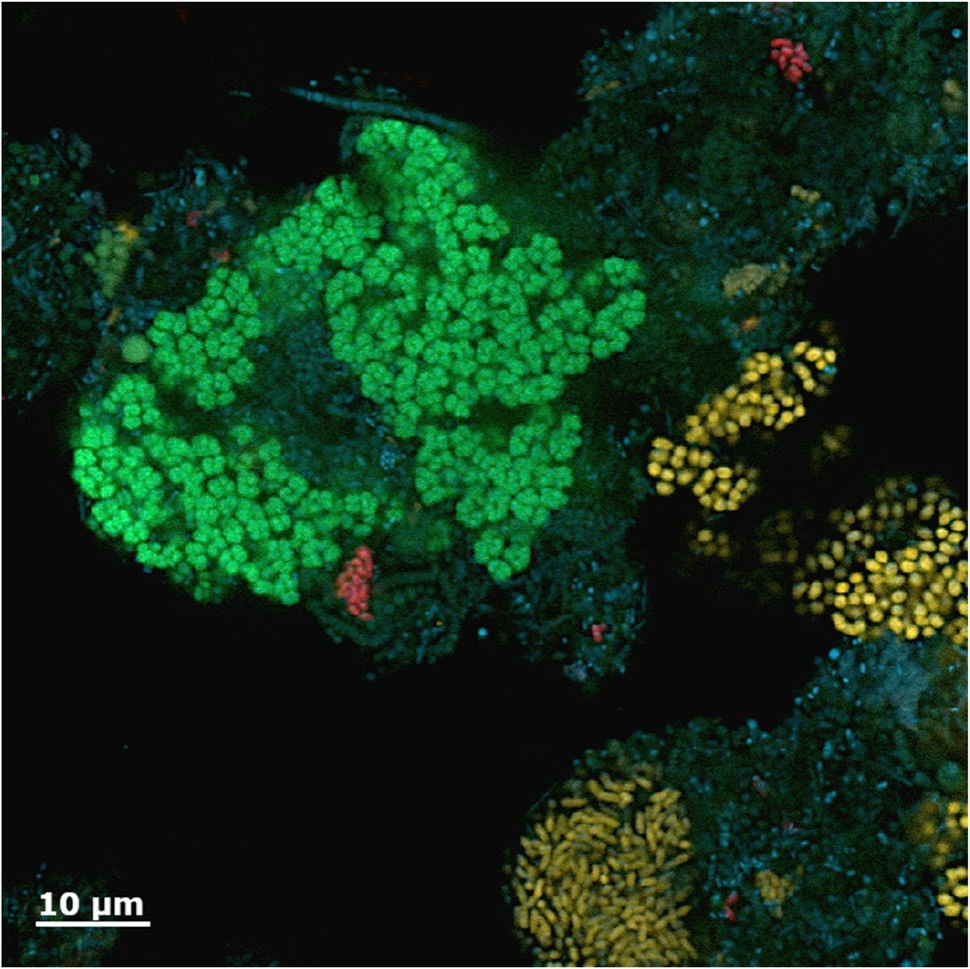
Microcolonies of *Cand.* Nitrotoga in activated sludge of the WWTP Hamburg-Dradenau, sampled in early spring. *Nitrotoga* cells were labeled by fluorescence in situ hybridization (FISH) with the oligonucleotide probe Ntg122 (Lücker et al. 2015), and appear as pleomorphic short rods in red. *Nitrospira* (green) was labeled with probes Nsp662 and Nsp712, *Nitrosomonas* (yellow) with probes Nm190 and Nm1225. All bacteria were staind with DAPI (blue). The image was taken with a Zeiss LSM 800

The occurrence of *Cand.* Nitrotoga is not restricted to environmental habitats with more or less low dissolved inorganic nitrogen concentrations, but it also proliferates in nutrient-rich wastewaters and activated sludge. This became apparent with cultivation of a potential new player in municipal N-removal systems. Incubations at 10–17 °C of activated sludge (AS) from a WWTP in Hamburg resulted in the selective enrichment of the second cultivated *Cand.* Nitrotoga representative with preference for low temperatures (Alawi et al. 2009). *Cand.* Nitrotoga was since then detected in many full-scale municipal WWTPs and laboratory or pilot-scale bioreactors operated with AS, but its occurrence seemed to be dependent on the geographic location (Table S1) with a clearly different distribution than *Nitrospira* (Cohen et al. 2019). Seasonal high abundances were observed in WWTPs located in cold or moderate climates (see below). In the WWTP in Hamburg, the cell numbers of *Cand.* Nitrotoga were rather low compared to *Nitrospira* (Fig. 3); however, labeled fatty acid profiles suggested that it was well metabolically active (Kruse et al. 2013a).

So far, *Cand.* Nitrotoga is the only known psychrotolerant NOB (Moyer et al. 2017) and the capability to oxidize nitrite also at temperatures too low for other cultured NOB broadens biotechnological applications in order to save energy for heating processes. Wastewaters in temperate climate zones are rarely above 20 °C (Dempsey 2017) and frequently undergo seasonal nitrification failure in winter, because a reduced water temperature below 13 °C reduces activity of most nitrifiers (Johnston et al. 2019).

### Recirculating aquaculture systems

Comparable to activated sludge, nitrification in biofilters of recirculating aquaculture systems (RAS) is mostly dominated by *Nitrospira* (Foesel et al. 2008; Keuter et al. 2011; Kruse et al. 2013b; Hüpeden et al. 2020) but especially in the Northern Hemisphere reports about occurrences as well as functional dominance of *Cand.* Nitrotoga are increasing. *Cand.* Nitrotoga occurred in high abundances (> 40%) in cold freshwater and brackish RAS (8per mill salt) in Norway (Navada et al. 2019, 2020a). It was also the main NOB in a cold freshwater RAS in Northern Germany, which was operated at a slightly acidic pH of 6.8 (Hüpeden et al. 2016). Furthermore, *Nitrospira* and/or *Cand.* Nitrotoga were the most abundant NOB in freshwater as well as brackish RAS bioreactors in Finland (Pulkkinen 2020). In the freshwater system, their distribution seemed dependent on the type of bioreactor (probably with different oxygenation of the biofilms, see below), because *Nitrospira* (as NOB and comammox) dominated the fixed bed reactor, whereas *Cand.* Nitrotoga was most abundant in the moving bed reactor. From the biofilters of a marine RAS running with North Sea water, the first marine cultures of this NOB could be enriched and further characterized (Keuter et al. in preparation). Its role in nitrification in this RAS however seemed minor. With the relatively high occurrence of *Cand.* Nitrotoga in non-saline WWTPs, it is rather surprising that the cold-adapted NOB was not found in more freshwater aquaculture systems. Its presence in marine systems however seems less likely, given that in general, 16S rRNA gene sequences of *Cand.* Nitrotoga in marine settings were rarely detected (Boddicker and Mosier 2018).

## Lessons learned from *Cand.* Nitrotoga cultures

### Temperature adaptation

**Fig. 4:**
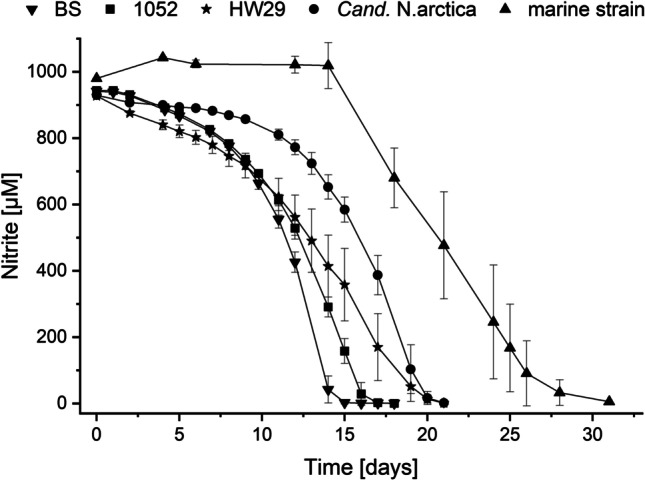
Nitrite oxidation of five *Cand.* Nitrotoga strains at an incubation temperature of 4 °C. The consumption of about 1 mM nitrite took between 15 (strain BS) and 30 (marine strain) days. Values are means ± standard deviations (error bars) for three biological replicates. *Cand.* N. arctica and *Cand.* Nitrotoga sp. 1052 originate from permafrost soil (Alawi et al. 2007; Keuter et al. in preparation), strain BS from activated sludge (Wegen et al. 2019), strain HW29, and the marine strain from aquaculture biofilters (Hüpeden et al. 2016; Keuter et al. 2017)

Low temperature is a main selective factor responsible for the dominance of *Cand.* Nitrotoga (Alawi et al. 2009; Lücker et al. 2015) and nearly all cultures grow well at 4 °C, in contrast to *Nitrospira* and *Nitrobacter* (Ushiki et al. 2013; Nowka et al. 2015b; Fig. 4). *Cand*. Nitrotoga arctica grows best between 10 and 17 °C and for most other strains of these NOB temperature optima between 17 and 22 °C were determined (Alawi et al. 2007; Hüpeden et al. 2016; Wegen et al. 2019; Ishii et al. 2017, 2020; Keuter et al. in preparation). An exception is the isolate *Cand*. Nitrotoga fabula from a WWTP in Austria, which is not adapted to low temperatures and only poorly grew < 20 °C (optimum at 24–28 °C) (Kitzinger et al. 2018). Interestingly, the isolate with a bean-like shape revealed a different morphology than other cultivated *Cand.* Nitrotoga strains, and the 16S rRNA gene sequence forms a sublineage in the phylogenetic tree. In accordance, the ANI (average nucleotide identity) values between *Cand*. Nitrotoga fabula and other cultures of this genus are rather low (Keuter et al. in preparation).

### Influence of the substrate concentration

At first, the new genus of NOB seemed to be already inhibited by relatively low substrate concentrations, especially in comparison to *Nitrobacter* (Alawi et al. 2007; Bartosch et al. 1999). Meanwhile, new additional data from cultivated strains are available and higher tolerance levels were found in correlation with the findings of Lücker et al. (2015). Three *Cand.* Nitrotoga strains (from marine aquaculture, coastal sediments, and *Cand*. Nitrotoga fabula) were resistant to > 20 mM, whereas cultures obtained from permafrost soils were nitrite-sensitive and did not grow in > 4 mM (Ishii et al. 2020; Kitzinger et al. 2018; Keuter et al. in preparation).

### Biochemistry

The key enzyme of nitrite oxidation, the nitrite oxidoreductase (NXR), is a molybdopterin-binding enzyme within the DMSO reductase type II family (Meincke et al. 1992; Lücker et al. 2010), which occurs in two different forms. The *Nitrobacter*-type NXR is bound to the inner side of the cytoplasmic and intracytoplasmic membranes (Spieck et al. 1998) and is closely related to nitrate reductases (NarG) of denitrifying bacteria (Kirstein and Bock 1993). The *Nitrospira*-type NXR faces the periplamic space and is related to the NXR in anaerobic ammonium-oxidizing *Brocardiaceae* (Lücker et al. 2010). Mostly, the substrate affinity of NOB with a periplasmic NXR (*Nitrospira*, *Nitrospina*) is higher in comparison to NOB with a cytoplasmic localization (*Nitrobacter*, *Nitrococcus*, *Nitrolancea*), and the inhibition threshold for nitrite is lower (Off et al. 2010; Nowka et al. 2015a).

*Cand.* Nitrotoga contains a new type of periplasmic nitrite oxidoreductase, which is phylogenetically distinct to the key enzymes with the same localization (Kitzinger et al. 2018; Boddicker and Mosier 2018) and K_m_ values for nitrite (measured with total cells) revealed values between those of *Nitrospira* and *Nitrobacter*. In detail, the K_m_ was in a moderate range of 25–89 µM nitrite for the different species of *Cand.* Nitrotoga (Ishii et al. 2017; Kitzinger et al. 2018; Nowka et al. 2015a; Wegen et al. 2019) and therefore presents rather a *K*-strategist (like *Nitrospira*) than an *r*-strategist (like *Nitrobacter*). The maximum specific activity of *Cand.* Nitrotoga (V_max_ = 19–52 µmol nitrite/mg protein per hour) lies in the *Nitrospira* range, but is clearly lower than the oxidation rates of *Nitrobacter* (Kitzinger et al. 2018; Nowka et al. 2015a; Wegen et al. 2019).

### Carbon fixation and adaptation to high DO

With regard to CO_2_ fixation, *Cand.* Nitrotoga differs from the other NOB with periplasmic NXR by using the Calvin-Benson-Bassham (CBB) cycle like *Nitrobacter*, *Nitrococcus*, and nitrite-oxidizing *Chloroflexi* (Boddicker and Mosier 2018; Kitzinger et al. 2018; Sorokin et al. 2012; Spieck et al. 2020b). This cycle requires a higher energy input than the reductive TCA cycle used by *Nitrospira* and *Nitrospina* (Berg 2011). Another concern is that some enzymes of the rTCA cycle are oxygen-sensitive, and therefore, this cycle is widespread among anaerobic or microaerobic bacteria. Its functioning in aerobic bacteria like *Nitrospira* requires enzymatic adaptations (Lücker et al. 2010). In contrast, the enzymes of the CBB cycle have a high robustness to molecular oxygen (Berg 2011), which can effect a competitive advantage over *Nitrospira* in high-oxygen habitats, as was shown for *Nitrobacter* (Downing and Nerenberg 2008; Huang et al. 2010). Nevertheless, in accordance with their microaerophilic ancestors, *Cand.* Nitrotoga as well as *Nitrospira* can cope with low DO since at least some species have a high affinity cytochrome cbb_3_ oxidase (Bayer et al. 2021; Boddicker and Mosier 2018; Kitzinger et al. 2018).

Apart from the enzymatic equipment, it is speculated that lipid patterns are involved in protection from high DO like regulation by hopanoid abundance in some NOB, but not in *Cand.* Nitrotoga (Elling et al. submitted).

### Low pH values

Two strains of *Cand.* Nitrotoga, N. arctica and HW29, were highly enriched from slightly acidic environments and had low pH optima of 6.4 and 6.8, respectively. Sustaining the reduced pH during cultivation, *Nitrospira* was finally eliminated from *Cand.* Nitrotoga (Hüpeden et al. 2016; Wegen 2017). It seemed therefore that low pH was another selective factor for the competition between *Cand.* Nitrotoga and *Nitrospira*. However, tests for pH preferences of other *Cand.* Nitrotoga cultures showed that the pH optimum lies in the neutral to slightly alkaline range as typical for nearly all NOB (Ishii et al. 2020; Kitzinger et al. 2018). Further examples from bioreactors revealed that *Cand.* Nitrotoga neither grew better in pH of 6.4 than in 7.4, nor could it dominate over *Nitrospira* at low pH levels (Wegen et al. 2019). Accordingly, FISH-microautoradiography (MAR) of a nitrite-oxidizing enrichment showed that both NOB were active at a pH of 6.4 (Hüpeden 2016). Therefore, adaptation of *Cand.* Nitrotoga to low pH values cannot be generalized, and in summary, the pH value appeared less stringent for NOB selection than temperature.

### Influence of ammonium

Some NOB of the phylum *Chloroflexi* (*Nitrolancea*, *Cand.* Nitrocaldera) turned out to require an external N-source for assimilation, because genes for the reduction of nitrite to ammonium are missing (Sorokin et al. 2012; Spieck et al. 2020a, b). Similarly, reproducible growth of *Cand.* Nitrotoga sp. BS and AM1 could only be recovered by addition of ammonium (Ishii et al. 2017; Wegen et al. 2019). Ammonium auxotrophic *Cand.* Nitrotoga cells might have adapted to metabolic networks in nature, where nutrients are exchanged in bacterial communities (Pande and Kost 2017). This way, *Cand.* Nitrotoga sp. AM1P and BS could save energy for nitrite reduction to ammonium (Ishii et al. 2017). Other *Cand.* Nitrotoga representatives were not stimulated by ammonium and can grow on nitrite as sole nitrogen source (Boddicker and Mosier 2018; Kitzinger et al. 2018; Keuter et al. in preparation).

In *Cand.* Nitrotoga cultures, the tolerance limit for ammonium was 30–40 mM (Ishii et al. 2017; Wegen et al. 2019). Although this NOB could neither be found by FISH nor by specific PCR primers in reactor types receiving a very high ammonium influent as present for example in animal rendering (Lücker et al. 2015), in wastewater short-term experiments (24 h), *Cand.* Nitrotoga survived in the presence of 1150 mg L^−1^ total ammonia nitrogen (TAN) (≙ 82 mM) (Li et al. 2020). In comparison, these concentrations are far below the activity inhibition value of ammonium measured for *Nitrobacter* (Hunik et al. 1993).

### Alternative metabolism

Organic matter or other substrates are important if they are suitable to accelerate or replace growth on nitrite. The genome of *Cand*. Nitrotoga fabula contains a complete pathway for hydrogen oxidation as possible alternative energy source (Kitzinger et al. 2018), but presents a less flexible metabolism comparable to *Nitrospira* (Koch et al. 2014, 2015). Apart from the capacity for lithoautotrophic growth with nitrite as substrate and CO_2_ as carbon source, *Cand.* Nitrotoga strains can use simple organic substances as known for other NOB (Steinmüller and Bock 1976; Spieck et al. 2006). The nitrite oxidation rate was stimulated by pyruvate and lactate (Ishii et al. 2020) or acetate and dextrose (Boddicker and Mosier 2018) and *Cand*. N. fabula has a transporter system for branched amino acids (Kitzinger et al. 2018). Yet, it is not clear if the activity increase results from mixotrophic growth, or from hydrogen peroxide detoxification as known for AOA (Kim et al. 2016; Ishii et al. 2020). A study of Yi et al. (2019) investigated the response of the microbial community to changes in soil nutrients and found that *Cand.* Nitrotoga abundance was strongly positively correlated with total nitrogen, further suggesting the use of an elevated concentration of substrate and/or of organic matter released by the degradation of complex substances. In addition, genome annotations of *Cand.* Nitrotoga strains revealed that they carry genes for sulfite oxidation, which might be used as alternative energy source (Boddicker and Mosier 2018; Kitzinger et al. 2018). The use of sulfur compounds correlates with the metabolic repertoire of other NOB (Füssel et al. 2017; Lücker et al. 2013; Palomo et al. 2018; Starkenburg et al. 2008).

Urea is an important dissolved organic N-compound in domestic wastewater (Hanson and Lee 1971) and RAS, where it may be 12–13% of the total dissolved nitrogen excreted by fish (Dalsgaard et al. 2015). The capacity for cleavage of urea was found in most *Nitrospira* (except *N. defluvii)* (Koch et al. 2015), but not in *Cand.* Nitrotoga (Boddicker and Mosier 2018; Kitzinger et al. 2018), supporting an advantage of the former NOB in the systems for water treatment.

Furthermore, *Cand.* Nitrotoga was one of the most prominent bacterial taxa (up to 6.8% of total bacteria) in anoxic/oxic reactor systems fed with acetate or glucose (Xing et al. 2018; Li et al. 2019). However, a possible denitrifying potential and mixotrophic or heterotrophic growth of *Cand.* Nitrotoga still require proofs from further experimental studies.

## Competition of *Cand.* Nitrotoga with *Nitrospira* and *Nitrobacter*

The distribution of different NOB in the environment reflects the ecological niche differentiation based on distinct metabolic features. As a consequence, cultivation conditions have to be altered in order to meet requirements of specialized nitrifiers. For example, lowering the substrate concentration resulted in the enrichment of *Nitrospira* versus *Nitrobacter* from activated sludge and soils (Bartosch et al. 1999, 2002). Several other parameters that influence the composition of NOB communities were identified, like temperature, DO, pH, and salt (Alawi et al. 2009; Huang et al. 2010; Hüpeden et al. 2016; Navada et al. 2019). Since multiple environmental or operational parameters determine the composition of the NOB community, it is not easy to define conditions which support the dominance of *Cand.* Nitrotoga in a given habitat. Some parameters and applications which facilitated a high abundance of this NOB in water treatment are listed in Table 1 and table S1 and are discussed in the following sections. Screening of the literature was restricted to the name “*Nitrotoga*” which might lead to some bias in interpretation and underestimation of the global distribution, but enabled the most comprehensive view on these NOB.

**Table 1:** Critical parameters for selection of *Cand.* Nitrotoga over *Nitrospira* (condensed version of table S1)

Selective factor	Value	Molarity	Comment	Reference
Low temperature	< 17 °C		Most strains	Alawi et al. 2009
Low pH	5.5–6.8		Some strains	Hüpeden et al. 2016
High DO	1–3 mg L^−1^	31–94 µM	Lower affinity than *Nitrospira*	Zheng et al. 2020
High nitrite	4.2–420 mg N L^−1^	0.3–30 mM	Lower affinity than *Nitrospira*	Lücker et al. 2015; Nowka et al. 2015a
High FA	220 mg N L^−1^	15.7 mM	Less inhibited than *Nitrospira*	Li et al. 2020
High FNA	1.8 mg N L^−1^	0.13 mM	Less inhibited than *Nitrospira*	Ma et al. 2017
High sulfide	< 20 mg S L^−1^	< 0.6 mM	More resistant than *Nitrospira*	Delgado Vela et al. 2018

### Low temperature

The nitrifying community in most WWTPs consisted of *Nitrospira* as main NOB (Daims et al. 2006). Nevertheless, in a screening of WWTPs in Central Europe, Scandinavia, and North America, *Cand.* Nitrotoga turned out to belong to the core community and outcompeted *Nitrospira* in some of the plants (Lücker et al. 2015; Chen et al. 2020; Saunders et al. 2016). Its relative 16S rRNA gene sequence abundance can reach 0.5–2% or even 4% (Johnston et al. 2019; Numberger et al. 2019; Saunders et al. 2016; Kruglova et al. 2020). In reactor systems for the treatment of ammonia-contaminated airstreams operated at 10 °C, *Cand.* Nitrotoga constituted the only detected NOB (Gerrity et al. 2016). As mentioned above for natural habitats, its abundance often varied seasonally and temperature variations were identified as main environmental factor for niche occupation (Liu et al. 2021; Zhao et al. 2020). The highest cell numbers of *Cand.* Nitrotoga were observed in late winter or in spring (Keene et al. 2017; Miłobędzka and Muszyński 2017; Numberger et al. 2019; Kruglova et al. 2020; Kim et al. 2021) when the temperature is still too low for optimal growth of other NOB. A temperature-dependent shift in the NOB community was confirmed in a bioreactor experiment with inorganic mine waters (Table S1; Karkman et al. 2011). Other bioreactor experiments revealed that strains of *Cand.* Nitrotoga are able to compete with *Nitrospira defluvii* at a temperature of 17 °C, but the abundance of *Cand.* Nitrotoga decreased when the temperature increased to 22 °C (Wegen et al. 2019).

A comparison of the seasonal nitrifying community between bench-scale and a lagoon WWTP in Canada revealed that the identified nitrifying bacteria (*Nitrosomonas*, *Nitrospira*, *Cand.* Nitrotoga) remained active even at low temperatures of 2–6 °C (Skoyles et al. 2020). Whereas *Nitrospira* dominated within the BioCord biofilm at bench-scale, *Cand.* Nitrotoga was more abundant in the field samples where the NOB had to withstand day and night cycles with alternating temperatures. Investigations about N_2_O emissions in water treatment confirmed the importance of *Cand.* Nitrotoga for low temperature nitrification (10–20 °C) during seasonal dynamics (Reino et al. 2017; Vieira et al. 2019; Gruber et al. 2021).

However, microautoradiography of activated sludge showed activity of *Cand.* Nitrotoga in a broad range from 4 to 27 °C (Lücker et al. 2015), which indicated that the function of these NOB is not restricted to low temperature, and, as mentioned above, not all *Cand.* Nitrotoga strains are cold-adapted or even psychrotolerant. For instance, close relatives of mesophilic *Cand*. Nitrotoga fabula were less competitive with *Nitrospira* at cold conditions and seem to be responsible for nitrite oxidation in warm water (> 20 °C) N-removal systems as observed in China and Australia (Liu et al. 2021; Zheng et al. 2020; Petrovski et al. 2020; Table S1).

### Low DO

Oxygen supply in large-scale WWTPs is expensive and oxygen limitation is a strategy to save energy but also needed for systems using anammox (anaerobic ammonia oxidation). The half-saturation constants for oxygen are far lower for *Nitrospira* than for *Nitrobacter* (Blackburne et al. 2007; Dytczak et al. 2008); for *Cand*. Nitrotoga these kinetic coefficients were not yet produced. In a partial nitritation-anammox (PNA) system for the treatment of municipal wastewater in Sweden, both *Nitrospira* and *Cand.* Nitrotoga resisted operation at intermittent aeration (Gustavsson et al. 2020), which is founded in their adaptation to microaerophilic habitats (Boddicker and Mosier 2018; Lücker et al. 2010). However, in an SBR, *Cand.* Nitrotoga was found to have a lower affinity to dissolved oxygen than *Nitrospira* (Zheng et al. 2020; Table 1 and S1), and a stepwise increase of the DO level in a PNA reactor from 0.4 to 1.8 mg L^−1^ (13–56 µM) led to an increase of *Cand.* Nitrotoga versus *Nitrospira* (Qian et al. 2021), which confirmed the tolerance of *Cand.* Nitrotoga against DO mentioned above. A DO of 2–2.5 mg L^−1^ (= 62.5–78 µM) was evaluated as suitable condition for the out-selection of *Nitrospira* and *Cand.* Nitrotoga for partial nitrification-anammox (Jiang et al. 2018); therefore, intensive aeration has to be applied with caution when enrichment of *Cand*. Nitrotoga is required.

An experiment on artificial nitrifying biofilms in AS (17 °C) revealed that thickness has a strong influence on the composition of the microbial community with *Cand.* Nitrotoga being almost restricted to thin biofilms (50 µm), which can be fully oxygenated (Suarez et al. 2019). In contrast, *Nitrospira* also colonized a thick biofilm (400 µm), which contained completely anoxic regions, with the highest abundance detected in 200 µm depth. The biovolume fraction of *Cand.* Nitrotoga amounted to 2.7% in the 50 µm biofilms, but only 0.5% in the 400 µm biofilms (Piculell et al. 2016). These results demonstrate different responses to oxygen gradients of the NOB, as also shown by Zhang et al. (2018).

### High N-load

Based on different substrate affinities mentioned above, *Nitrospira* is able to compete under low nitrite conditions, whereas *Nitrobacter* and *Cand.* Nitrotoga might benefit from higher substrate concentrations (Nowka et al. 2015a). This kind of competition was investigated by Kinnunen et al. (2017), who found that *Cand.* Nitrotoga outcompeted *Nitrospira* in a biofilm community at increased nitrite loading (1 mg N L^−1^ = 70 µM) and *Nitrospira* dominated at a tenfold lower substrate concentration. Likewise, using marker lipids for NOB community analyses in activated sludge, the *Cand.* Nitrotoga-typical fatty acid 16:1 *cis*9 was labeled with ^13^C-bicarbonate at nitrite concentrations between 0.3 and 30 mM. In contrast, the fatty acid 16:1 *cis*11, which is characteristic for *Nitrospira defluvii*, showed ^13^C-incorporation in AS samples exposed to maximum 3 mM nitrite (Kruse et al. 2013a). In addition to the substrate affinity, it was found that the competition between *Nitrospira* and *Nitrobacter* is also driven by the dilution rate (Winkler et al. 2017), but data for the minimal hydraulic retention time of *Cand.* Nitrotoga are rare.

### FA and FNA

Free ammonia (FA) and free nitrous acid (FNA) are known to cause severe inhibition to numerous bioprocesses in WWTPs and are gainfully used to suppress NOB in order to enhance partial nitrification in combination with anammox (Kim et al. 2006; Wang et al. 2016; Zhou et al. 2011; Yu et al. 2021). The inhibition thresholds for FA and FNA differ between NOB genera and *Nitrobacter* was less inhibited than *Nitrospira* (Blackburne et al. 2007; Duan et al. 2019). *Cand.* Nitrotoga can grow in the presence of relatively high concentrations of FA (up to 220 mg NH_3_-N L^−1^ = 15.7 mM; Li et al. 2020), which can be used as selective factor for enrichment (Ishii et al. 2017). In accordance, *Cand.* Nitrotoga became the dominant nitrite oxidizer, while *Nitrospira* was inhibited in water treatment under exposure to FNA and FA (Li et al. 2020; Zheng et al. 2020; Wang et al. 2021; Table 1 and S1). Similar to *Nitrobacter*, which revealed a residual activity of 10% in the presence of 1.0 mg HNO_2_-N L^−1^ (71 µM) (Blackburne et al. 2007), *Cand.* Nitrotoga, but not *Nitrospira*, tolerated 1.87 mg HNO_2_-N L^−1^ (134 µM; at a pH of 6.0) (Ma et al. 2017).

## Inhibition of NOB in water treatment

Nitrite oxidation is a very sensitive process, which can cause a chaotic instability of the whole nitrification process (Graham et al. 2007). While this is unfavorable in conventional aerobic nitrification systems, a specific inhibition of NOB followed by accumulation of nitrite is the goal when partial nitritation-anammox is used. For this purpose, an anaerobic pretreatment (Kouba et al. 2017a) or sulfide addition combined with a FA shock (see above) were used as stressors (Seuntjens et al. 2018). Other relevant factors which can suppress NOB are intensive aeration, high nitrate (low water exchange), salt addition (by the influent water), or chemicals (industrial wastewaters). Since *Cand.* Nitrotoga might be less affected than *Nitrospira* (or other NOB), the influence of some of these factors is discussed in the following.

### Nitrate accumulation

Nitrate inhibition is of special interest in recirculating aquaculture systems, where no denitrification unit is installed. In accordance, nitrite oxidizers on biocarriers from marine and brackish RAS have shown decreased nitrification rates with increasing nitrate concentrations (Keuter 2011). Most *Cand.* Nitrotoga cultures oxidized nitrite with a reduced rate in the presence of 10–20 mM nitrate (Kitzinger et al. 2018; Wegen et al. 2019; Keuter et al. in preparation). This value is in the same range as those determined for *Nitrospira* lineage I + II from AS (Nowka et al. 2015b), but much less than, e.g., for a marine *Nitrospira* isolated from a RAS (Keuter et al. 2011), *Nitrospira moscoviensis* (Ehrich et al. 1995), or *Nitrobacter* (Hunik et al. 1993). Although these levels should not be reached in RAS for the well-being of the reared animals (Camargo et al. 2005), they are not uncommon, and thus can be a factor shaping the nitrifying communities, in the worst case reducing nitrifying potentials of the biofilters.

### Salt inhibition

Critical salinity changes are required in RAS, e.g., during the production of Atlantic salmon or in coastal wastewater collection systems, which are infiltrated by seawater (Kinyage et al. 2018). Salt inhibition of nitrification can also occur where municipal and industrial sewage is combined, and *Nitrobacter* and *Nitrospira* are known to resist osmotic stress (Hunik et al. 1993; Moussa et al. 2006; Qiu and Ting 2013).

The *Cand.* Nitrotoga strains in culture seem to tolerate only low salinity concentrations, even one isolated from coastal sediment (Ishii et al. 2017). Alone, a strain of *Cand.* Nitrotoga enriched from a biofilter of a marine RAS had optimal growth between 0.5 and 3% NaCl (Keuter et al. in preparation). Nevertheless, *Cand.* Nitrotoga was resistant towards salt-spiked water (1.5% NaCl) in bioreactor experiments (Karkman et al. 2011) and was the dominant NOB tolerating rising salinities up to 3.2% in freshwater moving bed bioreactors (Navada et al. 2019). It was concluded that *Cand.* Nitrotoga represents an important NOB in cold-water nitrifying systems with variable salinities (Navada et al. 2020a, b). These findings showed once again that physiological limits tested in cultures may differ from those in situ, and that *Cand.* Nitrotoga is underrepresented by the few strains in culture so far.

### Sulfide inhibition

Hydrogen sulfide is produced biologically from sulfate in sewers and in anaerobic niches within treatment plants (Delgado Vela et al. 2018), and is discussed as electron donor for denitrification. Since sulfide especially inhibits NOB, it is used to establish partial nitritation/anammox (Kouba et al. 2017b). Batch experiments revealed that sulfide inhibition of nitrite oxidation depends on the microbial community and a *Nitrospira*-rich community was more inhibited than a community containing *Cand.* Nitrotoga and *Nitrobacter* (Delgado Vela et al. 2018). After a treatment of 150 mg S L^−1^ (4.7 mM), *Cand.* Nitrotoga was more resilient than *Nitrospira* (Seuntjens et al. 2018) and a similarly high tolerance level (128 mg S L^−1^ or 4 mM) was reported for *Nitrobacter* (Sekine et al. 2020). Correspondingly, Maestre et al. (2009) investigated the bacterial community in a biotrickling filter treating high loads of H_2_S and found a *Cand.* Nitrotoga fabula*-*like 16S rRNA gene sequence cluster, which might belong to resistant NOB.

### Resistance against chemicals and reactive oxygen species

*Cand.* Nitrotoga have repeatedly been found at contaminated sites like polluted rivers, petrochemical, or antibiotics-contaminated wastewaters (Brümmer et al. 2003; Li et al. 2011; Song et al. 2020). Their abundance was low in oil sands tailing ponds (Ramos-Padrón et al. 2011) but they belong to the dominant genera in biodegradation of naphthenic acids in process waters of oil sands (McKenzie et al. 2014). Notably, Zeman et al. (2014) concluded that *Cand.* Nitrotoga might be a novel hydrocarbon degrader due to the high relative abundance of 42% after a 12-month incubation at 30 °C. Survival in contaminated environments might be facilitated by an array of antibiotic and metal resistance genes in these organisms (Boddicker and Mosier 2018; Kitzinger et al. 2018). Several antibiotics were tested on cultures, and used in isolation procedures of this NOB (Ishii et al. 2020). When the effect of chromium for N-removal of granular sludge was investigated, *Cand.* Nitrotoga was able to tolerate 5 mg Cr (VI) L^−1^ (96 µM) which otherwise has a strong negative impact on NOB (Zheng et al. 2018).

With respect to their genomic interior, *Cand.* Nitrotoga should be able to resist oxidative stress to a certain degree (Boddicker and Mosier 2018; Ishii et al. 2020). This might be of relevance for dewatering sewage or disinfection in RAS with H_2_O_2_ (Alasri et al. 1992; Yang et al. 2018), but detailed studies on cultures were not performed so far. On the other hand, a low-dose UVA irradiation was successfully applied as new approach to eliminate NOB (*Nitrospira* and *Cand.* Nitrotoga) for N-removal via anammox (Chu et al. 2020).

## Possible application of *Cand.* Nitrotoga in phosphorus removal?

To protect receiving waters from eutrophication, municipal wastewater treatment plants have to remove not only excess nitrogen but also phosphorus. The process relies on a diverse bacterial community, which is able to store phosphorus intracellulary (Lawson et al. 2015). A novel polyphosphate accumulating organisms in domestic sewage (NCBI Accession number AB247475) was found with a high level of 16S rRNA gene sequence identity (99.0%) to *Cand*. N. arctica 6680. The novel nitrite oxidizing betaproteobacterium was further detected in several biological phosphorus removal plants (Kong et al. 2007; Ji and Chen 2010; Keene et al. 2017) and represented the only NOB in nitrifying-denitrifying phosphorus-accumulating granules in an activated sludge system operated at 12 °C (Figdore et al. 2018). Furthermore, both, *Cand.* Nitrotoga and *Nitrospira*, were found in low numbers in an enhanced biological phosphorus removal bioreactor (EBPR, 13–20 °C), but only *Cand.* Nitrotoga remained active during 120 days of operation with large changes of operational parameters across the different bioreactor redox zones (Lawson et al. 2015). These findings hint to a possible involvement of *Cand.* Nitrotoga in the phosphorus cycle and genes for phosphorus storage were found in some *Cand.* Nitrotoga strains (Boddicker and Mosier 2018; Ishii et al. 2020) as already known for other NOB. Additionally, electron-dense granules assumed to represent polyphosphate were observed in high numbers in cells of *Cand.* Nitrotoga in activated sludge (Alawi et al. 2009). Whether *Cand.* Nitrotoga have indeed a function in phosphorus removal in water treatment warrants further attention.

In general, NOB are survivalists supported by the storage of diverse reserve material. *Nitrobacter* uses polyphosphate as metabolic buffer (Eigener and Bock 1972) and C-storage compounds like poly-ß-hydroxybutyrate (PHB) and glycogen (van Gool et al. 1971). Glycogen deposits are present in all NOB, which was confirmed by genomic analyses for *Cand.* Nitrotoga (Kitzinger et al. 2018; Ishii et al. 2020). Notably, the combined removal of nitrogen and phosphorus with C-storage (PHA) at low temperature and participation of *Cand.* Nitrotoga seems promising at low C/N ratio (Yang et al. 2019).

## Microdiversity of *Cand.* Nitrotoga populations

In two reported cases, different strains of *Cand.* Nitrotoga have been enriched from the same source material (Ishii et al. 2020; Wegen et al. 2019), and these strains appeared to be adapted to differing nitrite concentration or temperatures. This could be an example of ecological niche separation in *Cand.* Nitrotoga, similar to previous findings in *Nitrospira* (Maixner et al. 2006). Diversity within the genus based on niche differentiation is still a new avenue for *Cand.* Nitrotoga-focused research, but experiments that used contrasting substrate conditions let us suggest that respectively different *Cand.* Nitrotoga strains are active in the same habitat (Kruse et al. 2013a; Lücker et al. 2015). Molecular surveys of biofilm communities in RAS or flow-through microcosms could differentiate between several representatives of *Cand.* Nitrotoga and the resident strain could not be replaced by invaders of the same genus despite their low phylogenetic distance (Navada et al. 2020a; Kinnunen et al. 2018). The coexistence of multiple NOB, even if they are closely related, supports stabilization of the system in case of disturbances (Santillan et al. 2020).

## Conclusion

The perception that nitrite oxidation at low temperature is mainly driven by highly specialized bacteria of *Cand.* Nitrotoga was confirmed by cultivation-based studies, metagenomic surveys, and biotechnological experiments. The phylogenetic distinct nitrite oxidoreductase points to a separate evolution event, and despite the same orientation in the periplasmic space, *Cand.* Nitrotoga and *Nitrospira* differ in their substrate affinity and carbon fixation pathway. Consequently, these competitors can be separated by selected nitrite concentrations and elevated DO, which can be used to stabilize low-temperature nitrification processes. In terms of inhibition of NOB for partial nitrification/anammox, the robust *Cand*. Nitrotoga is highly resistant against treatment with FA and FNA, exposure to sulfide, and toxicants and survives harsh conditions similar to *Nitrobacter*. Therefore, *Cand.* Nitrotoga is a suitable candidate for nitrite oxidation under stress as necessary for the treatment of industrial sewage.

In contrast to other NOB, *Cand.* Nitrotoga occupies its own physiological niche of low temperature and is therefore an important NOB for N-removal in natural and engineered ecosystems which are influenced by seasonal temperature fluctuations. It often is not permanently prevalent like *Nitrospira*, but rather reveals a “bloomy” distribution when conditions are advantageous in competition with other NOB.

In future, genome analyses might give further hints for additional physiological capacities of this NOB. Identification of the promoting factors for growth of *Cand.* Nitrotoga (e.g., elevated CO_2_ concentrations, organic matter, iron) provides the background for advanced nitrogen removal techniques. Especially cycling between anaerobic and aerobic incubation for enhanced biologically phosphorus removal (EBPR) is a worthwhile matter of future research.

## Supplementary Information

Below is the link to the electronic supplementary material.

Supplementary file1 (PDF 349 KB)
