# Supplementary material for: Relevance of Candidatus Nitrotoga for nitrite oxidation in technical nitrogen removal systems
Source: Appl Microbiol Biotechnol. 2021 Sep 11;105(19):7123–39. doi: 10.1007/s00253-021-11487-5 (PMC8494671; doi:10.1007/s00253-021-11487-5)
Supplement: Supplementary file 1 — Supplementary file1 (PDF 349 KB) [file 253_2021_11487_MOESM1_ESM.pdf]

Applied Microbiology and Biotechnology

Relevance of *Cand.* Nitrotoga for nitrite oxidation in technical nitrogen removal systems

Eva Spieck[1*], Simone Wegen[1], Sabine Keuter[1]

[1]Department of Microbiology and Biotechnology, Universität Hamburg, Germany

*Corresponding author:

e-mail: eva.spieck@uni-hamburg.de

telephone: +49 (0) 40 42816 424

Table S1 Overview of operational conditions involved in niche separation of *Cand.* Nitrotoga in technical systems

Main factor	Value	Process	Geographical location	Abundance of *Cand.* Nitrotoga (and other NOB)	Other factors/ methods	Reference
Tempera-ture	10 °C	BR,WW	Finland	SBR 0.3 %, MBR 0.6 %	synthetic WW	Kruglova et al. 2020
	seasonal	WWTPs		highest in spring (up to 4-5 %)	temperature 7-15 °C (spring), 17-22 °C (summer)	
	5 °C	BR, lab-scale	Finland	only NOB, coexistence with *Nitrobacter* when salt-spiked	mine waters	Karkman et al. 2011
	10 °C	BR, pilot-scale		*Nitrospira* high abundant		
	15-16 °C	BR, RAS	Finland	*Nitrotoga* 0.86 %, *Nitrospira* 1.12 %	freshwater	Pulkkinen 2020
				Nitrotoga 0.26 %, *Nitrospira* 0.85 %	brackish water	
	seasonal	WWTP	Sweden	increase during cold season	bioaugmentation (7-15 °C)	Stenström and la Cour Jansen 2017
	13 °C	MBBR, PN-A	Sweden	coexistence together with *Nitrospira* and *Nitrobacter*	decreasing substrate (311–27 mg-N L^{-1} of ammonium)	Persson et al. 2017
	seasonal	WWTP	Denmark	core member, sometimes more abundant than *Nitrospira*	mean water temperature in summer 18 °C	Saunders et al. 2016
	16-19 °C	MBR, AS	Denmark	*Nitrotoga* 0.02-0.04 %, *Nitrospira* 3.6-4.4 %	DO 0.8-3.7 mg L^{-1}	Ziegler et al. 2016
	7-16 °C	(DIC-)SBR, AS	Germany, Austria, Switzerland	only NOB in 2 BR, coexistence with *Nitrospira* in other BR	municipal (and industrial)	Lücker et al. 2015

	seasonal	WWTP	Germany	highest abundance in April (max 1.19 %), *Nitrospira*: max 1.19 % in July	14-24 °C	Numberger et al. 2019
	seasonal	WWTPs	Poland	higher abundance in March than September (up to 1.9 %)	municipal (and industrial)	Miłobędzka and Muszyński 2017
				Nitrospira: higher abundance in September than March		
	5-10 °C	WWTP	The Netherlands	27 % of total clones after 4 days	reverse osmose membrane	Bereschenko et al. 2010
	seasonal	WWTPs, AS	Canada	core community, abundance varies with temperature	ambient temperature -3 to 22 °C, highest in July (1.6 %)	Chen et al. 2020
	seasonal	lagoon WWTP	Canada	*Nitrotoga* dominant in field, *Nitrospira* dominant in bench-scale	BioCord biofilm	Skoyles et al. 2020
	seasonal	SBR, AS	USA	core member, average relative abundance >0.5 %	10-20 °C	Johnston et al. 2019
	10-20 °C	BR	Spain (The Netherlands)	grows well at low temperature	granular sludge	Reino et al. 2017
	seasonal	WWTP	Portugal	correlation with N_2O production during winter	Frielas winter 19-20 °C	Vieira et al., 2019
	14 to 4 °C	BR, AS	China	stable occurrence	inoculum: *Nitrotoga+Nitrospira,* DO > 4 mg L^{-1}	Liu et al. 2021
	22 to 34 °C			decrease		
	16-26 °C	SBR, AS	China	high at ambient temperature	DO decrease, FA/FNA exposure	Zheng et al. 2020

	22-28°C	aeration tank, AS	China	same abundance as *Nitrospira* at normal and high ammonium loading	MAS (microaerobic activated sludge process)	Zhang et al. 2020
	seasonal	WWTP, SBR	Australia	high cell number during summer	9-25 °C, DO >1 mg L^{-1}	Petrovski et al. 2020
	10°C	sand filters	Denmark	more abundant than *Nitrospira*	groundwater, production of drinking water	Albers et al. 2018
	14-16°C	sand filter	USA	about 5% of clone library, *Nitrospira* dominated	treatment of drinking water	White et al. 2012
	<7 °C	industrial-scale BF	China	*Nitrotoga* 3.5 % (upper layer), *Nitrospira* up to 24 % (middle layer)	removal of iron, manganese and ammonia from groundwater	Zhang et al. 2018
	17° and 28 °C	MBBR, brackish RAS	Germany	only *Nitrospira* active, *Nitrotoga* and *Nitrobacter* detected	0.3, 3 and 10 mM nitrite	Kruse et al. 2013b
pH	6.0	BR	China	benefit for *Nitrotoga*, *Nitrospira* was negatively impacted	21 °C, FNA treatment	Ma et al. 2017
	6.8	MBBR, freshwater RAS	Germany	coexistence of *Nitrotoga* and *Nitrospira*	13 °C	Hüpeden et al. 2016
DO	1.0-3.0 mg O_2 L^{-1} (31-94 µM)	PN-A	Sweden	resisted intermittent aeration together with *Nitrospira*	winter (12-14 °C)	Gustavsson et al. 2020
	0.2 mg O_2 L^{-1} (6.2 µM)	EBPR	USA	lower abundance than *Nitrospira*	pilot scale	Keene et al. 2017
	>2 mg O_2 L^{-1} (>62.5 µM)			high abundance		

	1-3 to 0.4-0.7 mg O_2 L^{-1} (31-94 to 13-22 µM)	SBR, AS	China	lower affinity to DO than *Nitrospira*	23 °C	Zheng et al. 2020
	0.3 mg L^{-1} (9 µM)	BR, AS	Korea	not detected, *Nitrospira* of very low abundance	in combination with high FA (20 mg L^{-1})	Le et al. 2020
	anoxic	Anammox WW	China	withstand low DO, proliferated in large granules	anoxic granule reactor, 15-17°C	Liu et al. 2017
	2.5 mg L^{-1} (78 µM)	Anammox BR	China	*Nitrotoga* and *Nitrospira* were out-selected within 30 days	anammox immobilized in volcanic carriers	Jiang et al. 2018
	1.8 mg L^{-1} (56 µM)	CSTR, PN-A,	China	high enrichment under excessive aeration	granular sludge, low-strength ammonium wastewater, 20 °C	Qian et al. 2021
	fully oxygenated	MBBR, AS	Sweden	*Nitrotoga* in thin biofilms (50 µm)	biofilms on carriers, 17 °C	Suarez et al. 2019
	anoxic in deeper parts			*Nitrospira* also present in 400 µm thick biofilms (max at 200 µm depth)		
	increasing DO, decreasing DOM	pilot scale plant, WW	Hungary	increasing abundance of *Nitrotoga* and *Nitrospira* in the last elements of a cascade system	wastewater cascade system, 12 °C	Benedek et al. 2014
Nitrite	1 mg HNO_2-N L^{-1} (70 µM)	silicone tubes	Denmark	*Nitrotoga* outcompeted *Nitrospira* in the biofilm	23 – 25 °C, tap water flow-through biofilm system	Kinnunen et al. 2017
	0.1 mg HNO_2-N L^{-1} (7 µM)			*Nitrospira* dominated		
	0.3-30 mM	WWTP	Germany	significant labeling of 16:1 *cis*9 (major lipid of *Nitrotoga*)	FAME-SIP, 17 and 28 °C	Kruse et al. 2013a
	0.3-3 mM			significant labeling of 16:1 *cis7* and 16:1 *cis*11 (specific for *Nitrospira*)		

FNA/FA	1.87 mg HNO_2-N L^{-1} (134 µM)	BR	China	*Nitrotoga* survived, *Nitrospira* not	pH 6.0, 21 °C	Ma et al. 2017
	1.78 mg HNO_2-N L^{-1} (127 µM)	SBR, AS	China	dominant NOB	pH 5.5, 22 °C, DO 4 mg L^{-1}	Zheng et al. 2020
	153 mg NH_3–N L^{-1} (10.9 mM)			dominant NOB	pH 9.0	
	220 mg NH_3-N L^{-1} (15.7 mM)	SBR, AS	China	highly resistant, *Nitrospira* and *Nitrobacter* were inhibited	22 °C	Li et al. 2020
	5-12 mg NH_3–N L^{-1} (0.36-0.86 mM)	PN	China	more abundant than *Nitrospira*	granular sludge, 25 °C	Liang et al. 2015
Salinity	increase rate 0-15 ‰ d^{-1}	MBBR	Norway	dominant NOB, tolerated salinities up to 32 ‰	12 °C, biocarrier from freshwater RAS	Navada et al. 2019
Sulfide	150 mg S L^{-1} (4.7 mM)	batch tests/MBR	Belgium	*Nitrotoga* highly resistant, *Nitrospira* was inhibited	15°C	Seuntjens et al. 2018
				Nitrospira more abundant	combination with FA shock (30 mg L^{-1}, 1 h)	
	20 mg S L^{-1} (0.6 mM)	batch tests	USA	*Nitrotoga* and *Nitrobacter* are more resistant than *Nitrospira*	anaerobic-anoxic-oxic, 25 °C	Delgado Vela et al. 2018
Phosphorus	12 °C	SBR, AS	USA	only NOB	phosphorus accumulating granules	Figdore et al. 2018
	13-20 °C	EPBR pilot plant	Canada	potentially active in all redox zones	anaerobic, anoxic, aerobic	Lawson et al. 2015

Abbreviations: BR = bioreactor
SBR = sequence batch reactor
MBR = membrane bioreactor
MBBR = moving bed bioreactor
EBPR = enhanced biological phosphorus removal
CSTR = continuous stirred tank reactor
WW = wastewater
AS = activated sludge
WWTP = wastewater treatment plant
PN = partial nitritation
A = anammox
BF = biofilter
DIC = differential internal cycling
SRT = solid retention time
FAME-SIP = fatty acid methyl ester-stable isotope probing

References:

Benedek T, Táncsics A, Szilágyi N, Tóth I, Farkas M, Szoboszlay S, Krifaton C, Hartman M, Kriszt B (2014) Analysis of biofilm bacterial communities responsible for carbon removal through a reactor cascade treating wastewater. World J Microbiol Biotechnol 30:977–987. https://doi.org/10.1007/s11274-013-1516-9

Bereschenko LA, Stams AJM, Euverink GJW, van Loosdrecht MCM (2010) Biofilm formation on reverse osmosis membranes is initiated and dominated by *Sphingomonas* spp. Appl Environ Microbiol 76:2623–2632. https://doi.org/10.1128/AEM.01998-09

Le LT, Lee S, Bui XT, Jahng D (2020) Suppression of nitrite-oxidizing bacteria under the combined conditions of high free ammonia and low dissolved oxygen concentrations for mainstream partial nitritation. Environ Technol Innov 20:101135. https://doi.org/10.1016/j.eti.2020.101135

Liang Y, Li D, Zeng H, Zhang C, Zhang J (2015) Rapid start-up and microbial characteristics of partial nitrification granular sludge treating domestic sewage at room temperature. Bioresour Technol 196:741–745. https://doi.org/10.1016/j.biortech.2015.08.003

Liu W, Yang D, Chen W, Gu X (2017) High-throughput sequencing-based microbial characterization of size fractionated biomass in an anoxic anammox reactor for low-strength wastewater at low temperatures. Bioresour Technol 231:45–52. https://doi.org/10.1016/j.biortech.2017.01.050

Persson F, Suarez C, Hermansson M, Plaza E, Sultana R, Wilén BM (2017) Community structure of partial nitritation-anammox biofilms at decreasing substrate concentrations and low temperature. Microb Biotechnol 10:761–772. https://doi.org/10.1111/1751-7915.12435

Stenström F, la Cour Jansen J (2017) Impact on nitrifiers of full-scale bioaugmentation. Water Sci Technol 76:3079–3085. https://doi.org/10.2166/wst.2017.480

Zhang X, Li S, Zheng S, Duan S (2020) Impact of dissolved oxygen and loading rate on NH_3 oxidation and N_2 production mechanisms in activated sludge treatment of sewage. Microb Biotechnol 14:419–429. https://doi.org/10.1111/1751-7915.13599

Ziegler AS, McIlroy SJ, Larsen P, Albertsen M, Hansen AA, Heinen N, Nielsen PH (2016) Dynamics of the fouling layer microbial community in a membrane bioreactor. PLoS One 11:e0158811
